# Presence of ear lobe crease may predict intermediate and high-risk patients with acute non-ST elevation acute coronary syndrome

**DOI:** 10.34172/jcvtr.2020.30

**Published:** 2020-08-25

**Authors:** Muzaffer Kahyaoglu, Cetin Gecmen, Ozkan Candan, Murat Gucun, Ahmet Karaduman, Ahmet Guner, Ender Ozgun Cakmak, Emrah Bayam, Yusuf Yilmaz, Mehmet Celik, Ibrahim Akin Izgi

**Affiliations:** ^1^Gaziantep Abdulkadir Yuksel State Hospital, Gaziantep, Turkey; ^2^Kartal Kosuyolu Heart and Research Hospital, Istanbul, Turkey; ^3^Umraniye Education and Research Hospital, Istanbul, Turkey

**Keywords:** Ear Lobe Crease, GRACE Score, NSTE-ACS

## Abstract

***Introduction:*** Ear lobe crease (ELC) was first described in 1973 as a physical examination finding indicating significant coronary artery disease (CAD). Several studies have been carried out in relation to this finding, and it has been shown that it is a marker of intima-media thickness, carotid artery disease, and CAD. We aimed to investigate the relationship between earlobe crease, which is a simple physical examination finding, and GRACE score as a risk estimation index in acute coronary syndromes without ST-segment elevation (NSTE-ACS) patients.

***Methods:*** 360 patients (mean age 62.2 years, 70% male) were included in our study. Patients were divided into two groups of GRACE scores ≤ 109 and >109, 167 patients were enrolled in group 1, and193 cases in group 2.

***Results:*** The group 2 patients were older, had higher systolic blood pressure (SBP) levels, a higher rate of hypertension, higher glucose levels, lower creatinine clearance levels, higher initial and peak troponin levels, lower hemoglobin levels, lower left ventricular ejection fraction (LVEF) and higher Gensini scores than the patients in group 1. The higher GRACE score group had markedly increased frequencies of ELC compared to the lower GRACE score group (80.8% vs. 24.5%, respectively, *P* < 0.001).

***Conclusion:*** The presence of ELC may predict moderate to high risk group of patients with NSTEACS.

## Introduction

Despite invasive treatments, acute coronary syndrome is one of the foremost causes of mortality even now.^1^ Unstable angina (UA), acute non-ST elevation myocardial infarction (NSTEMI), and myocardial infarction with acute ST elevation (STEMI) are the three presentations of acute coronary syndromes (ACS).^1^ After initial medical treatment, risk stratification of ACS patients is necessary for defining those with the highest risk for further cardiac events who can benefit from a more aggressive therapeutic approach.^2^ According to 2015 ESC guidelines for ACS without persistent ST segment elevation, it is recommended to use established risk scores for prognosis estimation with Class 1 recommendation.^1^

Based on the clinical trials performed, risk factors that could benefit from high risk and early invasive treatment have been determined and identified.^3-5^ Several validated risk prediction tools have been identified such as Thrombolysis in Myocardial Infarction (TIMI) risk score, the Global Registry of Acute Coronary Events (GRACE) Risk model, Crusade long-term mortality score, CHADS2 score and ACTION registry score, but according to the guidelines, the most commonly used ones are GRACE and TIMI risk scores.^6-8^ GRACE risk score is a detailed standard risk scoring system used for early diagnosis, risk classification, prognosis and treatment of ACS, and this risk score predicts the risks of hospital mortality and six-month mortality for all patients with ACS.^9^

Ear lobe crease (ELC) was first described in 1973 as a physical examination finding indicating significant coronary artery disease (CAD).^10^ Several studies have been carried out in relation to this finding, and it has been shown that it is a marker of peripheral arterial disease, carotid artery disease and increased carotid intima thickness.^11,12^ Based on these studies, we aimed to examine the interrelation between ELC, which is a simple physical examination finding, and GRACE score as a risk estimation index in patients with non-ST segment elevation acute coronary syndrome (NSTE-ACS).

## Materials and Methods

This study was planned as a single-center, non-randomized, prospective, and observational study. Seven hundred fifty-six patients who were diagnosed with NSTE-ACS according to the current guidelines were examined between September 2017 and April 2018. The patients were consecutively recruited in this study. Each one of the external ear disease or deformation, cardiogenic shock or hemodynamic instability, a previous history of CAD, concomitant moderate to severe valvular pathology, atrial fibrillation, ventricular arrhythmias, and atrioventricular block, active infection, acute hepatic and renal failure, cor pulmonale and serious pulmonary disease were used as the exclusion criteria. Two hundred sixty-two patients with STEMI or stable CAD, 6 patients with external ear disease, 68 patients with atrial fibrillation, 41 patients with severe valvular pathology, and 19 patients who met other exclusion criteria were excluded from the study. After exclusion, a total of 360 patients were included in our study.

**Figure 1:**
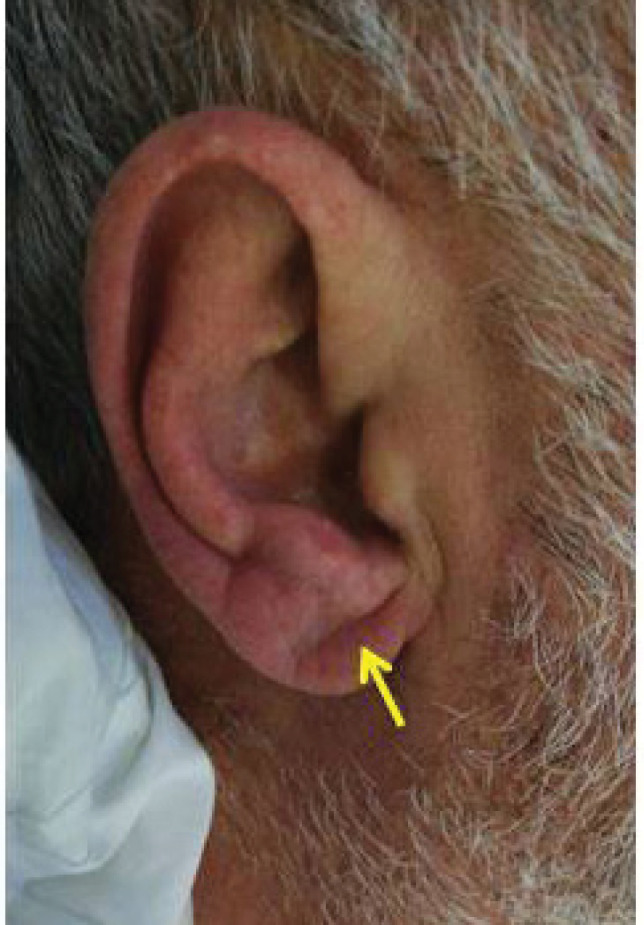
Diagonal ear lobe crease (arrow)

Visual examination was used for ELC determination to check for any major crease on the earlobe. If no major crease was seen on the ear lobes of either side, it was defined as ‘‘absence of ELC’’; if major crease was seen on at least one ear lobe, it was defined as ‘‘presence of ELC’’ (Figure 1).

Hemogram, troponin, lipid profile, and other biochemical parameters were analyzed from blood samples taken during admission. Fasting plasma glucose levels ≥126 mg/dL or plasma glucose levels ≥200 mg/dL at any time plus diabetic symptoms or 2^nd^-hour plasma glucose levels ≥200 mg/dL in oral glucose tolerance test or HbA1C levels ≥6.5 were used to define diabetes mellitus (DM). Patients were evaluated with echocardiography by experienced echocardiographers according to the recommendations of the European Association of Cardiovascular Imaging. Biplane Simpson’s method was used for determining the left ventricular ejection fraction (LVEF).^13^

To assess the risk of recurrent events using clinical information, the GRACE score was utilized. The GRACE 2.0 ACS Risk Calculator was used in our study to calculate the GRACE risk score, including the following eight prognostic factors: age, heart rate, systolic blood pressure (SBP) and Killip class or diuretic consumption, baseline creatinine level or a history of chronic renal failure, ST-segment deviation, elevation in cardiac necrosis biomarkers and admission cardiac arrest.^6^ When NSTE-ACS patients were evaluated for whether they would receive invasive treatment, the GRACE score was included in the risk stratification assessment according to the current guidelines.^1^ Patients with GRACE scores > 140 are considered to be at high risk; therefore, early invasive strategies (<24 hours) are recommended for treatment.^1^ Patients with a GRACE score of > 109 and < 140 are considered to be in the moderate-risk group and invasive strategies (<72 hours) are recommended for these patients.^1^ GRACE scores <109 indicate low risk and a non-invasive stress test is recommended in this group of patients to detect inducible ischemia so that it could be decided whether to follow an invasive strategy.^1^ Based on this information, in our study, NSTE-ACS patients were divided into two groups as patients with GRACE scores ≤109 and those with GRACE scores >109 to evaluate the association between the presence of ELC and GRACE score levels. It was also evaluated whether any presence of this association would allow for the decision to perform an invasive strategy or a non-invasive treatment option in these two groups of patients. With reference to these values, patients were categorized into two groups as GRACE scores ≤109 and >109.

Gensini scoring system was used to determine the severity of CAD. Gensini score assessment was calculated according to stenosis severity as 1 point for <25% stenosis, 2 points for 26%–50% stenosis, 4 points for 51%–75% stenosis, 8 points for 76%–90% stenosis and 32 points for total occlusion.^14^ The score was then multiplied by a factor representing the importance of the position of the lesion in the coronary artery system.

### Statistical analysis

Variables are expressed as mean ± standard deviation or median (25^th^-75^th^ percentile) or as percentage. Conformity to normal distribution was measured using the Kolmogorov-Smirnov test and nonparametric tests were used to compare non-normally distributed variables. Student’s *t* test or Mann-Whitney U-test was used for comparisons of continuous variables and Chi-squared test was used to compare the distributions of categorical variables. Multivariate analysis was applied to significant parameters (*P* ≤ 0.05) in univariate analysis. Finally, independent predictors of high GRACE scores were stated using multiple logistic regression analyses. All statistical analyses were performed on SPSS v. 16.0 (SPSS, Inc., Chicago, IL). *P* values below 0.05 were considered statistically significant.

## Results

Three hundred sixty patients were included in our study. Three hundred sixty patients were divided into two groups according to their GRACE scores as ≤109 and >109. 167 subjects were put in group 1, and 193 cases were put in group 2. Clinical, laboratory, echocardiographic and angiographic findings of the patients in group 1 and group 2 are presented in Table 1. There were no statistically significant differences between the two groups with respect to percentages of DM, body mass index, heart rate levels, diastolic blood pressure, white blood cell count and low-density lipoprotein (LDL) cholesterol levels. Group 2 was older of age, had higher SBP levels, higher percentages of hypertension, higher glucose levels, lower creatinine clearance levels, higher initial and peak troponin levels, lower hemoglobin levels, lower LVEF and higher Gensini scores than group 1 (All *P* < 0.05). Group 2 showed higher percentages of ELC than group 1. The group with higher GRACE score had markedly increased frequencies of ELC compared to the group with lower GRACE score (80.8% vs. 24.5%, respectively, *P* < 0.001).

**Table 1:** Clinical, laboratory,echocardiographic and angiographic characteristics of study subjects

**Variable**	**All patients** **(N = 360)**	**Group 1 (GRACE score ≤109) (n = 167)**	**Group 2 (GRACE score >109) (n = 193)**	***P*** **value**
Age (y)	62.2±11.09	56.31±9.32	67.4±9.86	<0.001
Sex male (%)	252 (70%)	129 (77.2%)	123 (63.7%)	0.004
Diabetes mellitus	166 (46.1%)	72 (43.1%)	94 (48.7%)	0.17
Hypertension	205 (56.9%)	67 (40.1%)	138 (71.5%)	<0.001
BMI (kg/m^2^)	28.4 [26-31.2]	27.6 [26-31.1]	29.4 [26-31.2]	0.95
Heart rate (bpm)	81 [68.2-88.7]	82 [68-88]	80 [68.5-92]	0.502
Systolic blood pressure (mm Hg)	136 [121.5-158]	130 [121-151]	142 [121.5-166]	0.011
Diastolic blood pressure (mm Hg)	78 [70-89]	78 [70-89]	79 [70-89]	0.767
Glucose (mg/dL)	116.5 [90-163]	102 [90-144]	131 [93-167.5]	0.005
Creatinine clearance(mL/min)	82.9±25.06	92.86±19.5	74.3±26.1	<0.001
Hemoglobin (mg/dL)	13.6 [12.8-14.8]	14.2 [13-15.4]	13 [11.9-14.1]	<0.001
White blood cell	8.8±3.01	8.6±3.1	8.9±2.8	0.281
Initial troponin (ng/mL)	0.28 [0.05-1.05]	0.2 [0.04-0.8]	0.3 [0.08-1.21]	0.003
Peak troponin (ng/mL)	1.59 [0.64-6.8]	1.09 [0.38-3.1]	2.6 [1-8.29]	<0.001
LDL (mg/dL)	120.3±36.9	121.6±37.4	119.2±36.6	0.546
Presence of ear lobe crease	197 (%54.7)	41 (%24.5)	156 (%80.8)	<0.001
EF (%)	60 [55-65]	56 [36-61]	51 [21-61]	<0.001
Grace score	111 [85.2-122]	84 [74-98]	121 [116-134]	<0.001
Gensini score	24 [14-32]	16 [12-28]	32 [18-44]	<0.001

BMI, body mass index; EF, left ventricle ejection fraction; LDL, low density lipoprotein.

From another perspective, patients with ELC were older of age, had higher SBP levels, higher percentages of hypertension, lower heart rate, lower creatinine clearance levels, higher initial and peak troponin levels, lower hemoglobin levels, higher GRACE score levels, lower LVEF and higher Gensini scores than group 1 (All *P* < 0.05) (Table 2).

**Table 2:** Clinical, laboratory,echocardiographic and angiographic characteristics of study subjects with and without ear lobe crease

**Variable**	**All patients** **(N = 360)**	**ELC( -) (n = 167)**	**ELC( +) (n = 193)**	***P*** **value**
Age (y)	62.2±11.09	57.4±10.5	66.2±9.9	<0.001
Sex male (%)	252 (70%)	116 (71.2%)	136 (69%)	0.729
Diabetes mellitus	166 (46.1%)	60 (38.8%)	106 (53.8%)	0.001
Hypertension	205 (56.9%)	63 (38.6%)	138 (72.1%)	<0.001
BMI (kg/m^2^)	28.4 [26-31.2]	27.6 [26-32.4]	29 [26-31.1]	0.923
Heart rate (bpm)	81 [68.2-88.7]	84 [72-92]	78 [68-88]	0.002
Systolic blood pressure (mm Hg)	136 [121.5-158]	131 [120-155]	140 [124.5-164]	0.028
Diastolic blood pressure (mm Hg)	78 [70-89]	80 [70-91]	78 [70-87.5]	0.300
Glucose (mg/dL)	116.5 [90-163]	104 [90-162]	126 [91-164]	0.209
Creatinine clearance(ml/min)	82.9±25.06	92.2±20.2	75.2±26.1	<0.001
Hemoglobin (mg/dL)	13.6 [12.8-14.8]	14 [12.9-15]	13.3 [11.9-14.1]	<0.001
White blood cell	8.8±3.01	8.4±3.1	9.1±2.9	0.024
Initial troponin (ng/ml)	0.28 [0.05-1.05]	0.12 [0.04-0.65]	0.47 [0.16-1.24]	<0.001
Peak troponin (ng/ml)	1.59 [0.64-6.8]	0.9 [0.3-3]	2.6 [1.03-8.18]	<0.001
LDL (mg/dL)	120.3±36.9	119.6±36.5	120.9±37.4	0.734
Presence of ear lobe crease	60 [55-65]	65 [55-65]	55 [50-60]	<0.001
EF (%)	111 [85.2-122]	83 [74-104]	120 [112-132]	<0.001
Grace score	24 [14-32]	16 [12-30]	32 [16-42]	<0.001
Gensini score	24 [14-32]	16 [12-28]	32 [18-44]	<0.001

BMI, body mass index; EF, left ventricle ejection fraction; LDL, low density lipoprotein.

Before multivariate analysis, correlation analysis was performed to check for multicollinearity, and a weak positive correlation was found between glucose and Gensini score (r: 0.193, *P* < 0.001). No significant correlation was found between glucose and hemoglobin or LVEF.

The presence of ELC, glucose level, hemoglobin level, LVEF, Gensini score and percentages of the male sex were examined with multivariate logistic regression test. Although age, troponin and creatinine clearance were significant in univariate analysis, these are not included in the multivariate analysis because of the parameters of the GRACE risk model. In the multivariate logistic regression analysis, presence of ELC (odds ratio [OR] = 8.17, 95% CI = 4.590-14.550, *P* < 0.001), hemoglobin level (OR = 0.737, 95% CI = 0.630-0.911, *P* = 0.003), LVEF (OR = 0.94, 95% CI = 0.901-0.982, *P* = 0.006), and Gensini score (OR = 1.045, 95%, CI = 1.015-1.076, *P* = 0.003) were detected as independent predictive parameters for high GRACE score (Table 3).

**Table 3:** The result of multivariate logistic regression analysis for the prediction high Grace score

**Variable**	**OR**	**CI**	***P***
Glucose	1	0.995-1.005	0.961
Hemoglobin	0.759	0.633-0.910	0.003
EF	0.940	0.901-0.981	0.005
Gensini score	1.046	1.022-1.071	<0.001
Sex male	1.499	0.746-3.013	0.256
Precence of ear lobe crease	8.163	4.587-14.526	<0.001

## Discussion

In this study, we observed that the presence of ELC, LVEF, hemoglobin level and Gensini score had independent predictive values for higher GRACE score in patients with NSTE-ACS. As far as we know, this is the first study reporting that the presence of ELC is associated with high GRACE score in this patient population.

LVEF, hemoglobin levels and Gensini score have been reported to predict high risk ACS patients in previous studies. In the IMMEDIATE study, it has been shown that LVEF, which was measured during hospitalization, could predict one-year death or hospitalization from heart failure in patients with ACS.^15^ As a parameter of ACEF score (Age, creatinine, ejection fraction), LVEF is used to show one-year mortality in patients with ACS undergoing percutaneous intervention.^16^ It has been shown that echo parameters such as LVEF added to GRACE and TIMI scores, which are used for risk stratification, increase the prognostic value of these risk scores.^17,18^ In a study by Liu et al, it was found that one-year mortality decreased with increased hemoglobin levels in STEMI patients.^19^ In a study of pooled data of ACUITY and HORIZONS-AMI studies, hemoglobin and anemia were detected to be independent predictors of prognostic information. The extent and severity of CAD may be assessed by Gensini score or a number of diseased vessels. Gensini score was positively and significantly associated with the GRACE score. Previous studies have shown that the severity of CAD increases with high GRACE score.^20,21^ In our study, the hemoglobin level, LVEF and Gensini score were determined as independent predictors for high GRACE score, similar to the above-mentioned studies.

Central obesity, Achilles tendon thickness, xanthoma, acanthosis nigricans and skin tag are important findings in atherosclerosis, and they have been used in determining high risk of atherosclerosis, such as CAD.^22,23^ ELC is a wrinkle-like line that extends from the tragus along the lobule to the posterior edge of the ear’s auricle and was first described in 1973 as a physical examination finding indicating significant CAD.^10^ Since its first description, there have been many other reports about ELC being a risk factor for ischemic heart disease.^24-26^ In addition, in postmortem studies, a positive correlation was shown between coronary artery stenosis and ELC.^27,28^ ELC occurs more frequently with increasing age, but it has been shown in many studies that it has an independent predictive value in demonstrating atherosclerosis independent of age.^29,30^

When the pathophysiological mechanism between ELC and CAD is examined, it is stated that myocardium and ear lobe are supplied by end-arterioles of the same genetic origin and therefore may be associated with atherosclerosis.^24^ Also, other studies have shown that the relationship between ELC and CAD may be related to elastin and elastic fiber loss. Previous studies have been clinically and pathologically examined, and degeneration of elastin, tearing of elastic fibers, and pre-arteriolar wall thickening have been detected in patients with ELC.^31^ In this way, the relationship with CAD can be established.

In addition to CAD, the presence of ELC in other atherosclerotic vascular diseases was investigated. Celik et al have shown an association between increased carotid IMT and diagonal ELC in healthy individuals during a standard health check-up.^12^ Shrestha et al detected that ELC was remarkably associated with markers of atherosclerotic changes in the arteries, such as plaque score and plaque number, as well as the carotid artery IMT in patients clinically indicated for ultrasound examination for carotid arteries.^32^ In a study conducted with ABI on peripheral arterial diseases, patients with ELC had lower levels of ankle-brachial index than those without ELC, and a significant relationship was found between the presence of ELC and the severity of peripheral artery disease.^33^

Various studies have shown not only the relationship between the presence of ELC and the presence of CAD but also a correlation with the extent and severity of CAD, regardless of cardiovascular risk factors. In their study, Elliott and Karrison proposed that diagonal ELC was related with increased all-cause and cardiac mortality.^34^ Patients with ELC had a higher risk of atherosclerotic heart diseases such as cardiac death, non-fatal myocardial infarction, or coronary artery bypass surgery. In addition, Tranchesi Júnior et al compared patients with angiographically documented CAD to a control group, and they showed a remarkable relationship between the presence of ELC and severity of CAD as measured by the number of major diseased arteries.^35^ Another study performed by Hou et al investigated the effect of ELC on clinical outcomes in a patient group undergoing coronary artery stent implantation. Hou et al showed that, in patients who underwent coronary angiography and had >4 risk factors, the number of diseased vessels and Gensini scores was significantly higher in patients with bilateral ELC than those without ELC and those with unilateral ELC. Also, the incidence of major adverse cardiovascular events was higher in patients who underwent coronary stent implantation in the ELC group (OR=5.568, 95% CI = 1.059-29.273, *P* = 0.028).^36^ In another study conducted by Shmilovich et al, computed tomography angiography showed that the presence of ELC was associated with significant CAD, multivessel disease and number of segments with plaque.^37^

After diagnosis and initial medical therapy, rapid risk classification in patients with ACS is necessary to detect patients with the highest risk of further cardiac events who may benefit from a more aggressive therapeutic approach.^2^ Risk classification is performed using approved risk prediction models that contain the most important predictors of the outcome. The GRACE risk score is a validated score for risk classification in patients with ACS, derived from a multicenter registry. The GRACE risk score is a better prognostic tool than the TIMI risk score and PURSUIT score.^38^ ELC has previously been shown as an independent risk factor for the presence and extent of CAD and cardiovascular mortality and morbidity in many studies. However, the relationship between ELC and risk stratification tools used in ACS has not been investigated. Our study showed that the existence of ELC has an independent predictive value for higher GRACE score in NSTE-ACS. The presence of ELC may predict moderate and high-risk groups in NSTE-ACS patients.

Our study had some limitations. Single-center enrollment, lack of randomization, and lack of prognostic data due to the absence of clinical follow-up were the major limitations. Also, if we had more patients, we could have achieved more comprehensive results.

## Conclusion

ELC, which is a simple physical examination finding, may have prognostic value in evaluating NSTE-ACS patients, and patient groups with ELC may be evaluated as having higher risk. It is important for clinicians to keep in mind the presence of ELC, in addition to other parameters during physical examination in NSTE-ACS.

## Competing interests

None to declared.

## Ethical approval

Ethics committee approval was received for this study from the Ethics Committee of the Health Sciences University Kartal Kosuyolu Heart and Research Hospital (Decision Number: 2017/2/102).

## Funding

None.
